# Exercise Strategies for Optimizing Aerobic Capacity and Skeletal Muscle Performance in Older Adults

**DOI:** 10.1093/geroni/igaa057.1703

**Published:** 2020-12-16

**Authors:** Dallin Tavoian, David Russ, Brian Clark

**Affiliations:** 1 Ohio University, Athens, Ohio, United States; 2 University of South Florida Morsani College of Medicine, Tampa, Florida, United States

## Abstract

Most older adults do not exercise regularly. Among those who do, the majority only perform one type of exercise, and— as such— are either not getting the benefits of endurance exercise or resistance exercise. The aim of this pilot study was to determine which standalone exercise strategy has the greatest effect on both cardiorespiratory and lower-extremity muscular function in insufficiently active older adults 60 to 75 years of age (N = 14). Participants were randomly assigned to either resistance training (RT, n=5), moderate intensity continuous training on a stationary bicycle (MICT, n=4), or high-intensity interval training on a stationary bicycle (HIIT, n=5) for supervised exercise sessions three times per week for 12 weeks. Maximal oxygen consumption increased a comparable amount in all groups (11.9±11.2% for HIIT vs. 8.0±14.8% for MICT vs 9.8±5.7% for RT). Leg extensor power did not change in the HIIT group (-0.34±5.2%), but increased by 5.2±9.7% in the MICT group and 14.5±26.1% in the RT group. Leg extensor strength decreased by 1.7±22.1% in the HIIT group and 0.6±6.4% in the MICT group, but increased by 27.3±21.2% in the RT group. These findings demonstrate that RT results in improved lower-extremity strength and power, as well as improvements in maximal aerobic capacity comparable to MICT and HIIT in older adults. Thus, RT should be promoted as an essential exercise strategy for older adults, particularly for individuals who are inactive or that are only performing one type of exercise regularly.

